# Serum AIM2 as a Prognostic Biomarker for Disease Severity and Delirium in Acute Pancreatitis: A Multicenter Prospective Cohort Study

**DOI:** 10.1002/brb3.71712

**Published:** 2026-08-20

**Authors:** Shiyang Dong, Bo Zhang, Yidong Jing, Shaojun Zhang, Zihao Tang, Jianye Wu, Yujun Rao

**Affiliations:** ^1^ Emergency Department Shengzhou People's Hospital (Shengzhou Branch of the First Affiliated Hospital of Zhejiang University School of Medicine, the Shengzhou Hospital of Shaoxing University) Shengzhou Zhejiang People's Republic of China; ^2^ Department of Hepatobiliary Surgery Shengzhou People's Hospital (Shengzhou Branch of the First Affiliated Hospital of Zhejiang University School of Medicine, the Shengzhou Hospital of Shaoxing University) Shengzhou Zhejiang People's Republic of China; ^3^ Department of Gastroenterology Lishui City Hospital of Traditional Chinese Medicine Lishui Zhejiang People's Republic of China; ^4^ Department of Gastroenterology Lishui Hospital of Wenzhou Medical University Lishui Zhejiang People's Republic of China

**Keywords:** absent in melanoma 2, acute pancreatitis, delirium, severity

## Abstract

**Objective:**

Absent in melanoma 2 (AIM2) is implicated in inflammation, and delirium is a common complication following acute pancreatitis (AP). We investigated whether serum AIM2 levels are related to severity and delirium after AP.

**Methods:**

This multicenter prospective cohort study included 349 patients with AP and 100 controls. According to a ratio of 7:3, patients were randomly assigned to study group (244 cases) and validation group (105 cases). Severity metrics included the acute physiologic and chronic health evaluation II (APACHE II), Ranson scale, computed tomography severity index (CTSI), and bedside index for severity in acute pancreatitis (BISAP). Severity correlation and delirium association were determined by using multifactorial analyses. Delirium‐prediction model was verified in the validation group.

**Results:**

Serum AIM2 levels were significantly higher in patients with AP than in controls. Serum AIM2 levels were independently correlated with the APACHE II scores, Ranson scores, BISAP scores, and CTSI scores. Serum AIM2 levels were linearly related to delirium risk after AP under the restricted cubic spline. Serum AIM2 levels, CTSI scores and BISAP scores were independently associated with delirium following AP. Serum AIM2 levels had predictive ability comparable to those of APACHE II, Ranson, BISAP, and CTSI scores. Delirium‐prediction model integrating serum AIM2 levels, CTSI scores, and BISAP scores was stable and clinically valid by using a series of statistical approaches, whether in the study group or validation group.

**Conclusions:**

Enhanced serum AIM2 levels are tightly correlated with disease severity and efficiently predict delirium following AP, suggesting that serum AIM2 may be a potential biomarker of AP.

## Graphical abstract

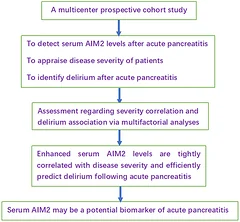

## 1 Introduction

Acute pancreatitis (AP), one of the most common acute inflammatory digestive diseases, is characterized by rapid progression and high rate of adverse clinical outcomes (Mederos et al. 2021). Pathophysiologically, auto‐digestion of pancreatic tissues and resultant pancreatic edema, hemorrhage, and necrotic injuries elicit local and even systemic inflammatory responses, thereby leading to multiple organ dysfunction and even failure (Lankisch et al. 2015). In clinical practice, the acute physiologic and chronic health evaluation (APACHE) II, Ranson scoring system, computed tomography severity index (CTSI), and bedside index for severity in acute pancreatitis (BISAP) are extensively used to assess AP severity (Zhou et al. 2019; Chauhan et al. 2022; Xu et al. 2023). Delirium, an acute cerebral dysfunction, is frequently encountered in patients with extracerebral syndromes including AP (Ye et al. 2024; Xu et al. 2019; Dong et al. 2020). Mechanistically, delirium is related to a variety of brain injury processes, including inflammation, oxidative reactions, brain ischemia, hypoxia, apoptosis, and others (Wilson et al. 2020; Keenan and Jain 2022). Considering that delirium can prolong hospitalization time, increase the risk of cognitive impairment, and promote morbidity and mortality, importance has been attached to its prediction (Jung et al. 2022; Körber et al. 2021; Kim et al. 2024). Moreover, blood samples are easily obtainable; therefore, blood markers have been highlighted with respect to their predictive implications in delirium during recent decades (Wang et al. 2024; Wan et al. 2020; Liu et al. 2022).

Inflammasomes are a group of pivotal complexes that extensively participate in the inflammatory processes of numerous disorders (Voet et al. 2019; Paerewijck and Lamkanfi 2022). Absent in melanoma 2 (AIM2), a crucial component of the inflammasome, is demonstrably present in brain tissues and is notably upregulated following acute brain injury (Fu et al. 2024; Li et al. 2021). AIM2 functions as a deleterious factor because its activation is experimentally associated with worsening brain edema, blood‐brain barrier disruption, increased neuronal death, and poor neurological functions (Yuan et al. 2020; Denes et al. 2015; Xu et al. 2021). Clinically, AIM2 is believed to be a biomarker of brain injury, given that blood AIM2 levels are strongly correlated with severity and adverse clinical outcomes in several brain injury diseases, such as acute cerebral infarction, traumatic brain injury, aneurysmal subarachnoid hemorrhage, and spontaneous intracerebral hemorrhage (Wang et al. 2021; Z. Chen, Zou, et al. 2024; Zhang et al. 2024; S. H. Chen, Hu, et al. 2024). In addition, AIM2 depletion substantially attenuates acinar cellular injuries, decreases systemic inflammation, and reduces the infiltration of macrophages/neutrophils in AP mice (Kang et al. 2016). Clearly, sterile brain injury is ascribed to systemic inflammation, oxidative stress, endothelial dysfunction, and organ dysfunction in the entity of extracerebral infection because pathogens are not detected in the central nervous system subjected to sepsis‐associated encephalopathy (Li et al. 2025). Since AIM2 participates in inflammatory amplification and sterile brain injury responses (Fu et al. 2024; Li et al. 2021), elevated circulating AIM2 may reflect systemic inflammatory burden and potentially contribute to delirium susceptibility. Taken together, serum AIM2 levels may be related to severity and delirium after AP. Currently, serum AIM2 levels were quantified to ascertain its relevance to post‐AP severity and delirium.

## 2 Materials and Methods

### 2.1 Study Design and Subject Enrollments

**FIGURE 1:**
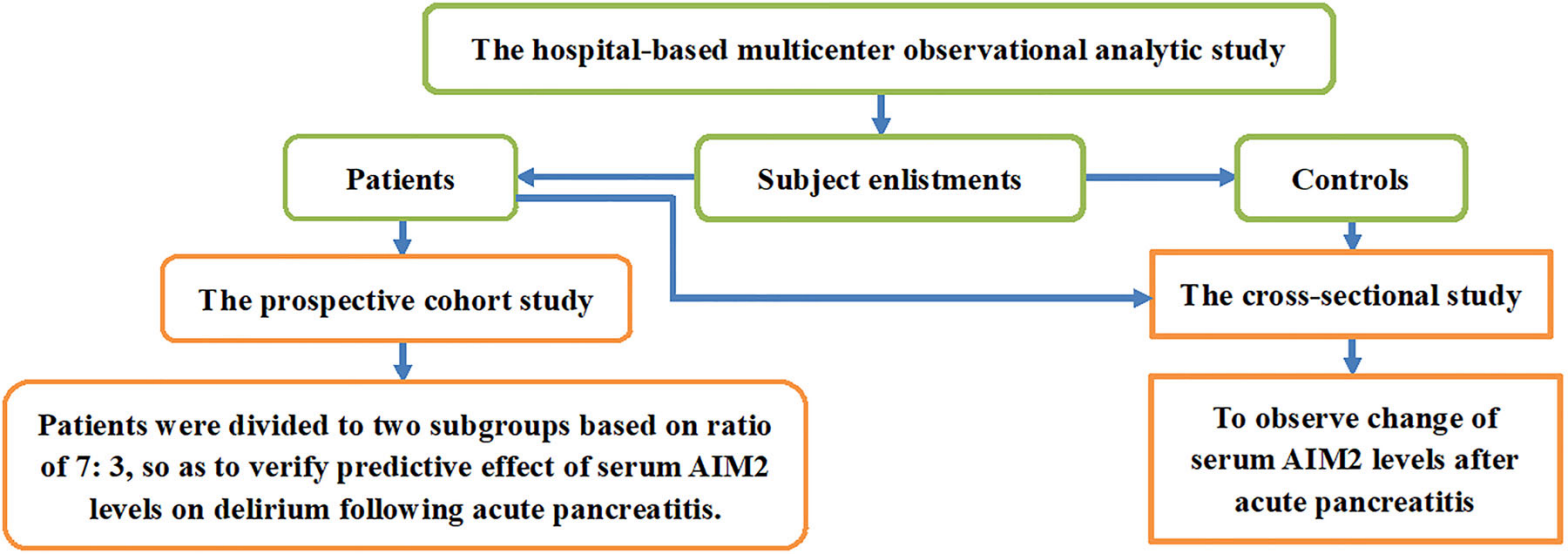
Diagrammatic sketch for study design. This observational study was divided into the cross‐sectional sub‐study and prospective cohort sub‐study, for the sake of investigating alteration of serum absent in melanoma 2 levels and their association with severity and delirium following acute pancreatitis. AIM2, absent in melanoma 2.

This multicenter observational study was conducted at the Shengzhou People's Hospital (Shengzhou, China), the Lishui City Hospital of Traditional Chinese Medicine (Lishui, China), and the Lishui Hospital of Wenzhou Medical University (Lishui, China) between January 2021 and January 2024. Inclusion criteria of AP patients included: (1) age of at least 18 years; (2) first‐in‐life pancreatitis; and (3) admission time not exceeding 24 h since pain. Exclusion criteria of AP patients encompassed: (1) histories of severe systemic diseases or other specific conditions, like uremia, cardiac failure, cirrhosis, myocardial infarction, pregnancies, malignancies, infections within recently a month, and surgeries within past six months and more; (2) histories of neuropsychiatric sicknesses, like stroke, moderate to severe traumatic brain injury, depression, Parkinson's disease, Alzheimer's disease, multiple sclerosis, intracranial tumors, and others; and (3) other situations, like missed visits, incomplete materials, rejection to participate, and unqualified samples. Controls were recruited from physical examinees at health examination centers of the above hospitals. The eligibility criteria of controls were as follows: (1) absence of some chronic diseases, but not including hypertension and diabetes mellitus; (2) normal results in some conventional tests, such as electrocardiogram, chest radiograph, blood cell counts, and so on. As displayed in Figure 1, this study encompasses two sub‐studies. In the cross‐sectional sub‐study, all controls and all patients were chosen to investigate changes in serum AIM2 levels after AP. In the prospective cohort study, all AP patients were randomly allocated to study group and validation group at a ratio of 7:3. Patients in the study group were selected to determine the role of serum AIM2 as a biomarker of delirium following AP. A model integrating independent predictors of delirium was constructed in the study group, and its clinical benefit was validated using patients from the validation group. The study was implemented in compliance with the regulations set forth in the Declaration of Helsinki, local laws, and institutional rules, and the Ethical Committees at the preceding three hospitals approved the study protocol (opinion numbers: LLW‐FO‐403, LW‐081, and SZRY‐ERC‐015). Following clarity about the study details, the patients’ legal proxies and controls independently signed informed consent forms.

### 2.2 Data Collections

Some baseline information of controls, including age, gender, height, body weight, alcohol drinking, tobacco smoking, hypertension, and diabetes mellitus, was documented. Body mass index (BMI) was calculated in accordance with the formulation as follows: body weight (kg) divided by height squared (m^2^). As for patients, some essential information, including age, sex, height, body weight, and time since the onset of pain, was collected. Several adverse lifestyle habits or chronic comorbidities, such as alcohol consumption, tobacco smoking, hypertension, and diabetes mellitus, were registered. AP etiologies were categorized into four categories: biliary, alcoholic, hypertriglyceridemic, and others. According to the revised Atlanta classification, AP was divided into three types: severe, moderately severe, and mild AP (Banks et al. 2013). Other severity indicators included APACHE II, Ranson scale, CTSI, and BISAP. Documented clinical, radiological and pathophysiological manifestations included multiple organ dysfunction syndrome, peripancreatic effusion, pancreatic necrosis, sepsis, acute respiratory failure, and acute renal injury. In‐hospital delirium of AP patients was assessed twice daily by using the confusion assessment method (CAM) (Ely, Inouye, et al. 2001; Ely, Margolin, et al. 2001). The assessors included physicians and nursing staff, who routinely participated in AP management, received standardized training regarding CAM application before study initiation and were blinded to serum AIM2 measurement results to minimize potential assessment bias.

### 2.3 Immune Analysis

Blood specimens from controls were collected via venipuncture at their entry into the study. Blood was drawn from patients at admission in the same manner. Blood samples were placed in tubes containing a gel separator, and the naturally coagulated blood samples were centrifuged at 3000 × *g* for 10 min. Supernatants were carefully aspirated and later transferred to Eppendorf tubes for storage in −80°C refrigerator, waiting for eventual testing. All samples were thawed to quantify serum AIM2 within three months of sampling to avoid protein breakdown. Serum AIM2 levels were measured using enzyme‐linked immunosorbent assay kit (Catalog No. BES3068K; Shanghai Bolsen Biotechnology Co. Ltd). The kit held a detection range of 0.156–10.0 ng/mL, and both intra‐ and inter‐batch variabilities were lower than 10%. Two repetitions of detections were performed for each sample by professional personnel who were shielded from the study details.

### 2.4 Statistical Analysis

Data analyses were performed using SPSS 25.0 (IBM Corp., Armonk, NY, USA). Qualitative data were presented as frequencies (percentages), and the chi‐square test and Fisher's exact test were used for intergroup comparisons. All quantitative variables were non‐normally distributed via the Kolmogorov‐Smirnov test, and subsequently were expressed as medians (percentiles 25^th^–75^th^); and the Mann–Whitney U test was applied for intergroup comparisons. Correlation of serum AIM2 levels with other variables was determined by the Spearman test. Subsequently, variables which were significantly correlated with serum AIM2 levels based on preliminary correlation analyses and those which were clinically relevant, were forced into the multivariable linear regression model to identify independently correlated factors. The variance inflation factor (VIF) was calculated to reflect multicollinearity, with VIF< 10 signifying non‐multicollinearity (Kim 2019). Restricted cubic spline analysis was performed to determine whether serum AIM2 levels were linearly associated with delirium risk. Variables showing significant associations on univariate analyses and those with clinical relevance were contained in the binary logistic regression model to identify independent predictors of in‐hospital delirium. Subgroup analysis was performed to determine the moderating effect of some conventional factors, such as age, sex, and BMI. E‐value was calculated for sensitivity analysis (Vale et al. 2021). The independent predictors of delirium were consolidated to create a model. The model was tested for discrimination efficiency, clinical benefit, stability, and improvement rate by performing receiver operating characteristic curve, decision curve, calibration curve analysis, as well as computing net reclassification improvement (NRI), and integrated discrimination improvement (IDI). In the validation group, the predictive effect of serum AIM2 levels on delirium was verified and the clinical value of the prediction model was demonstrated. Here, the sample size was estimated at Type 1 error value (alpha) of 0.05, test power (1‐beta) of 0.95, and Cohen's d index above 0.8, indicating an effect size in relation to serum AIM2 levels across delirium. By applying the G*Power 3.1.9.4 (Heinrich–Heine‐Universität Düsseldorf, Universitätsstraße 1, Düsseldorf, Germany), the priori power analysis was done to demonstrate the sufficient sample size. Missing data were assessed before statistical analysis. All variables included in the final analyses were completely available for enrolled participants; therefore, no missing value imputation was performed and complete‐case analysis was applied. Statistical significance was set at *p* < 0.05.

## 3 Results

### 3.1 Study Subject Features

A total of 414 patients with AP was initially appraised; 65 patients were afterwards excluded owing to histories of severe systemic diseases or other specific conditions (34 cases), histories of neuropsychiatric sicknesses (28 cases), missed visits (2 cases), and rejection to participate (1 case); and 349 patients were ultimately retained for clinical analysis. In addition, 100 controls participated in this study. As listed in Table 1, age, gender, BMI, alcohol consumption, cigarette smoking, hypertension, and diabetes were not statistically significantly different between patients and controls (all *p *> 0.05). In total, 244 and 105 patients were assigned to the study group and validation group respectively. The clinical, radiological, and biochemical data were not substantially different between the two subgroups (all *p* > 0.05; Table 2).

**TABLE 1:** Baseline features between controls and patients with acute pancreatitis.

Factors	All patients (*n = *349)	Controls (*n = *100)	*p* value
Age (years)	49 (39–59)	48 (42–61)	0.447
Gender (male/female)	201/148	60/40	0.667
Body mass index (kg/m^2^)	25.2 (22.3–27.4)	24.5 (22.0–26.2)	0.165
Alcohol drinking	116 (33.2%)	24 (24.0%)	0.079
Tobacco smoking	126 (36.1%)	26 (26.0%)	0.060
Hypertension	93 (26.6%)	19 (19.0%)	0.119
Diabetes mellitus	50 (14.3%)	7 (7.0%)	0.052
Admission time after pain (h)	13.9 (8.8–17.9)		
Sampling time after pain (h)	15.2 (10.2–19.5)		
Revised Atlanta classification (MAP/ MSAP/ SAP)	135/126/88		
Multiple organ dysfunction syndrome	80 (22.9%)		
Peripancreatic effusion	211 (60.5%)		
Pancreatic necrosis	77 (22.1%)		
Sepsis	73 (20.9%)		
Acute respiratory failure	70 (20.1%)		
Acute renal injury	30 (8.6%)		
Acute physiology and chronic health evaluation II scores	10 (4–15)		
Ranson scores	2 (1–3)		
Bedside index for severity in acute pancreatitis	2 (1–3)		
Computed tomography severity index	2 (1–3)		
Etiologies (biliary/ alcoholic/hypertriglyceremic/ others)	109/130/76/34		
Blood C‐reactive protein levels (mg/L)	37.4 (23.5–71.1)		
Blood leucocyte count (×10^9^/L)	13.8 (10.7–17.4)		
Blood procalcitonin levels (ng/mL)	2.7 (1.7–3.7)		
Blood calcium levels (mmol/L)	2.0 (1.9–2.2)		
Blood glucose levels (mmol/L)	10.7 (8.0–16.5)		
Blood creatinine levels (µmol/L)	113 (92–137)		
Blood urea nitrogen levels (mg/dL)	12.5 (9.4–15.5)		

*Note*: Qualitative variables were presented as counts (percentages) and quantitative variables were reported as medians (percentiles 25th‐75th). The statistical methods included the chi‐square test, Fisher exact test, t test and Mann–Whitney U test.

Abbreviations: MP, mild acute pancreatitis; MSP, moderately‐severe acute pancreatitis; SP, severe acute pancreatitis.

**TABLE 2:** Basiline features between study group and validation group in patients with acute pancreatitis.

Factors	Study group (*n = *244)	Validation group (*n = *105)	*p* value
Age (years)	48 (39–59)	49 (39–59)	0.875
Gender (male/female)	143/101	58/47	0.559
Body mass index (kg/m^2^)	25.2 (21.9–27.4)	25.6 (22.8–27.6)	0.323
Alcohol drinking	79 (32.4%)	37 (35.2%)	0.603
Tobacco smoking	89 (36.5%)	37 (35.2%)	0.825
Hypertension	62 (25.4%)	31 (29.5%)	0.425
Diabetes mellitus	32 (13.1%)	18 (17.1%)	0.325
Admission time after pain (h)	13.8 (9.0–17.8)	14.5 (7.4–18.1)	0.889
Sampling time after pain (h)	15.2 (10.3–19.4)	16.4 (8.8–19.6)	0.828
Revised Atlanta classification (MAP/ MSAP/ SAP)	97/89/58	38/37/30	0.624
Multiple organ dysfunction syndrome	53 (21.7%)	27 (25.7%)	0.416
Peripancreatic effusion	151 (61.9%)	60 (57.1%)	0.406
Pancreatic necrosis	52 (21.3%)	25 (23.8%)	0.606
Sepsis	51 (20.9%)	22 (21.0%)	0.991
Acute respiratory failure	49 (20.1%)	21 (20.0%)	0.986
Acute renal injury	21 (8.6%)	9 (8.6%)	0.991
Acute physiology and chronic health evaluation II scores	10 (5–16)	10 (4–14)	0.142
Ranson scores	2 (1–3)	2 (1–3)	0.120
Bedside index for severity in acute pancreatitis	2 (1–3)	2 (1–3)	0.818
Computed tomography severity index	2 (1–3)	2 (2–4)	0.160
Etiologies (biliary/ alcoholic/hypertriglyceremic/ others)	77/96/51/20	32/35/25/14	0.354
Blood C‐reactive protein levels (mg/L)	37.5 (24.3–69.7)	36.8 (22.1–71.1)	0.370
Blood leucocyte count (×10^9^/L)	14.1 (10.8–17.7)	13.7 (10.0–16.8)	0.335
Blood procalcitonin levels (ng/mL)	2.6 (1.8–3.8)	2.7 (1.7–3.6)	0.841
Blood calcium levels (mmol/L)	2.0 (1.9–2.2)	2.1 (1.9–2.2)	0.271
Blood glucose levels (mmol/L)	10.7 (8.2–15.8)	11.1 (7.6–17.3)	0.390
Blood creatinine levels (µmol/L)	115.7 (95.0–140.7)	109.0 (88.8–136.3)	0.189
Blood urea nitrogen levels (mg/dL)	12.6 (9.8–15.7)	11.9 (9.4–15.0)	0.145

*Note*: Qualitative variables were presented as counts (percentages) and quantitative variables were reported as medians (percentiles 25^th^–75^th^). The statistical methods encompassed the chi‐square test, Fisher exact test, t test and Mann–Whitney U test.

Abbreviations: MP, mild acute pancreatitis; MSP, moderately‐severe acute pancreatitis; SP, severe acute pancreatitis.

### 3.2 Variational Trends of Serum AIM2 Levels and Its Connection With Severity

**FIGURE 2:**
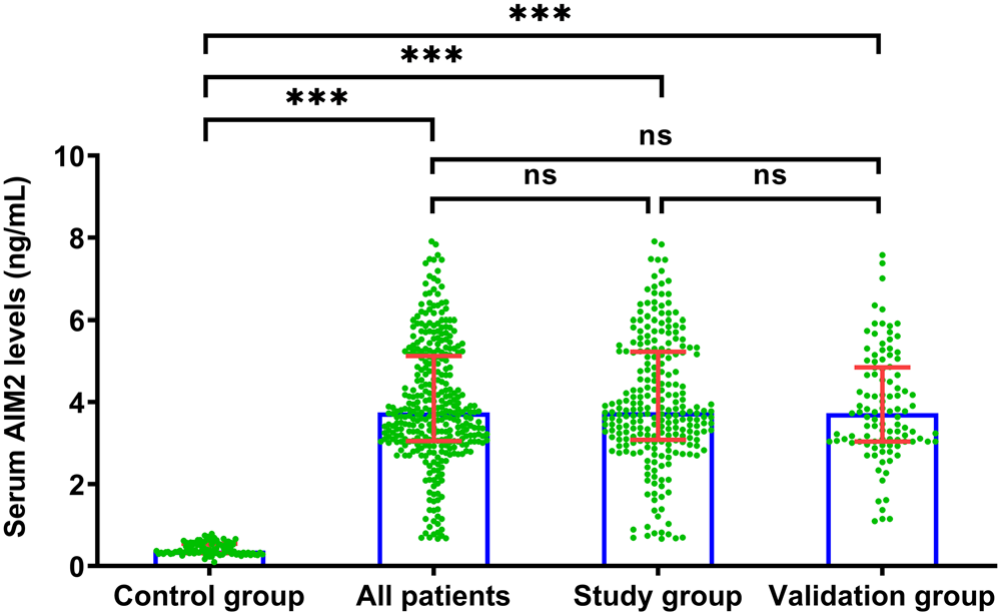
Alteration of serum absent in melanoma 2 levels after acute pancreatitis. Serum absent in melanoma 2 levels were not significantly different among three groups of patients (all *p* > 0.05). The levels were significantly higher in all groups of patients than in controls (all ^***^ *p* < 0.001). AIM2, absent in melanoma 2; ns, not significant.

**FIGURE 3:**
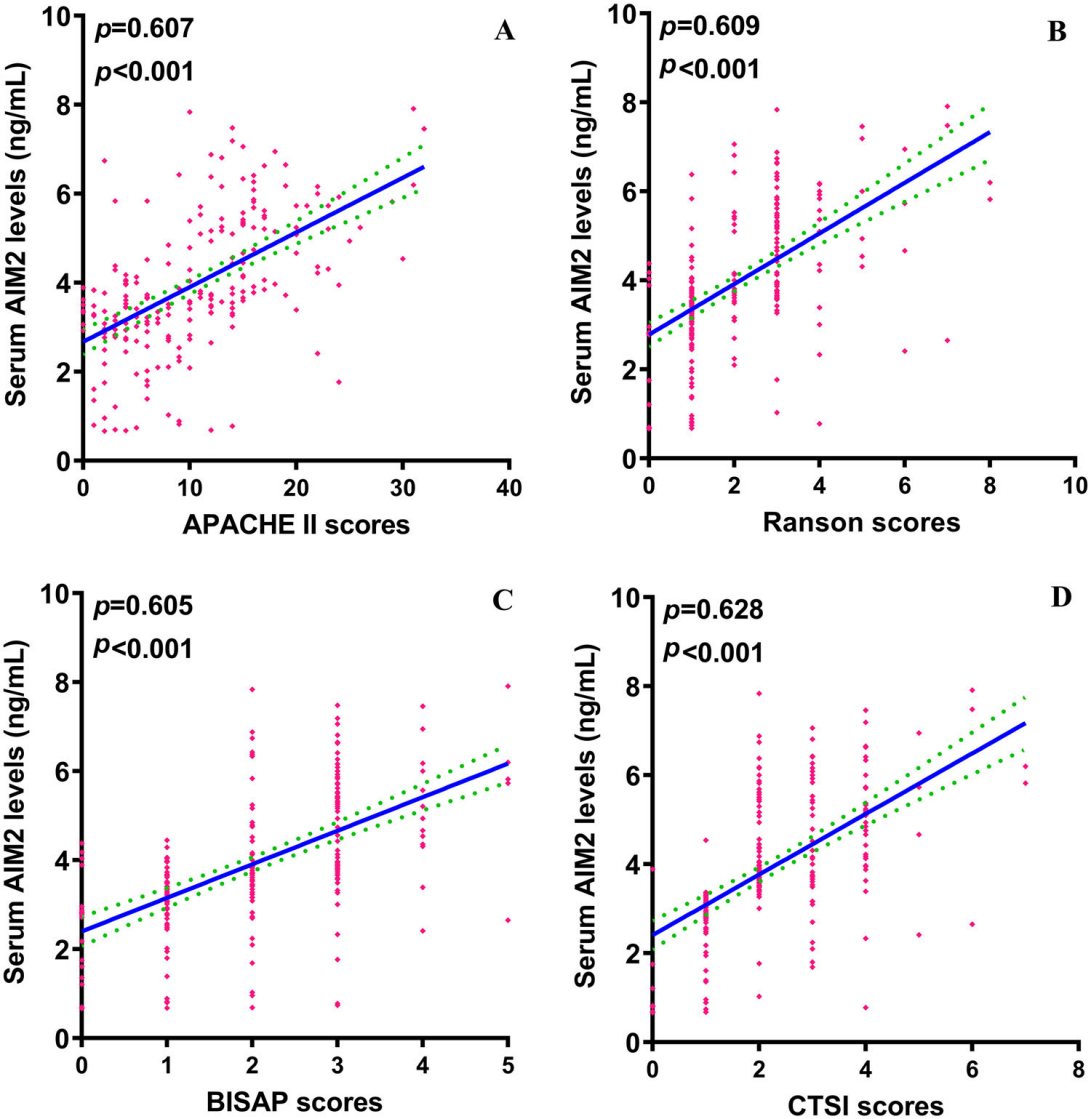
Relationship between serum absent in melanoma 2 levels and disease severity in the study group. Serum absent in melanoma 2 levels were significantly correlated with acute physiologic and chronic health evaluation II scores after AP (*p* < 0.001; A), Ranson scores (*p* < 0.001; B), bedside index for severity in acute pancreatitis scores (*p* < 0.001; C), and computed tomography severity index scores (*p* < 0.001; D). AIM2, absent in melanoma 2; APACHE II, acute physiological and chronic health evaluation II; BISAP, bedside index for severity in acute pancreatitis; CTSI, computed tomography severity index.

In Figure 2, serum AIM2 levels were not significantly different among all AP patients, and those patients in study group and validation group (all *p* > 0.05); and controls presented with substantially lower serum AIM2 levels than the above three groups (all *p* < 0.001). In the study group, serum AIM2 levels were significantly positively correlated with APACHE II scores (*p < *0.001; Figure 3A), Ranson scores (*p < *0.001; Figure 3B), BISAP scores (*p < *0.001; Figure 3C), and CTSI scores (*p < *0.001; Figure 3D). In addition to the above four severity indicators, the revised Atlanta classification, multiple organ dysfunction syndrome, peripancreatic effusion, pancreatic necrosis, sepsis, acute respiratory failure, acute renal injury, blood C‐reactive protein levels, blood leukocyte count, blood procalcitonin levels, blood glucose levels, and blood creatinine levels were closely correlated with serum AIM2 levels (all *p < *0.05; Table S1). The above‐mentioned significantly correlated variables and several clinically related metrics were inserted into the multivariate linear regression model, revealing that serum AIM2 levels were independently correlated with APACHE II scores, Ranson score, BISAP scores, and CTSI scores (all *p < *0.05; Table 3).

**TABLE 3:** Multivariate linear regression analysis of serum absent in melanoma 2 levels after acute pancreatitis in the study group.

Factors	Beta (95% CI)	*p* value	VIF
Age (years)	0.012 (−0.001–0.024)	0.060	1.109
Gender (male/female)	−0.307 (−0.893–0.278)	0.302	1.933
Body mass index (kg/m^2^)	0.033 (−0.011–0.078)	0.144	1.097
Alcohol drinking	0.182 (−0.249–0.613)	0.406	1.944
Tobacco smoking	0.314 (−0.134–0.763)	0.168	2.283
Hypertension	0.113 (−0.243–0.469)	0.532	1.174
Diabetes mellitus	0.198 (−0.280–0.676)	0.416	1.277
Admission time after pain (h)	0.218 (−0.059–0.496)	0.122	119.655
Sampling time after pain (h)	−0.198 (−0.466–0.069)	0.146	119.082
Revised Atlanta classification (MAP/ MSAP/ SAP)	0.252 (−0.012–0.515)	0.061	2.075
Multiple organ dysfunction syndrome	−0.094 (−0.576–0.388)	0.702	1.934
Peripancreatic effusion	−0.019 (−0.354–0.316)	0.911	1.295
Pancreatic necrosis	−0.198 (−0.737–0.340)	0.469	2.383
Sepsis	−0.180 (−0.671–0.312)	0.472	1.956
Acute respiratory failure	−0.179 (−0.635–0.278)	0.441	1.636
Acute renal injury	0.148 (−0.464–0.760)	0.634	1.442
Acute physiology and chronic health evaluation II scores	0.065 (0.025–0.106)	0.002	4.156
Ranson scores	0.276 (0.086–0.466)	0.005	5.624
Bedside index for severity in acute pancreatitis	0.355 (0.084–0.626)	0.011	5.492
Computed tomography severity index	0.299 (0.106–0.491)	0.002	3.048
Etiologies (biliary/ alcoholic/hypertriglyceremic/ others)	0.051 (−0.114–0.216)	0.545	1.137
Blood C‐reactive protein levels (mg/L)	0.003 (−0.001–0.007)	0.158	1.307
Blood leucocyte count (×10^9^/L)	−0.007 (−0.044–0.030)	0.716	1.418
Blood procalcitonin levels (ng/mL)	0.022 (−0.065–0.109)	0.619	1.161
Blood glucose levels (mmol/L)	0.025 (−0.006–0.056)	0.115	1.243
Blood creatinine levels (µmol/L)	−0.002 (−0.006–0.003)	0.390	1.221

Abbreviations: 95% CI, 95% confidence interval; MP, mild acute pancreatitis; MSP, moderately‐severe acute pancreatitis; SP, severe acute pancreatitis; VIF, variance inflation factor.

### 3.3 Serum AIM2 Levels and In‐Hospital Delirium Following AP

**FIGURE 4:**
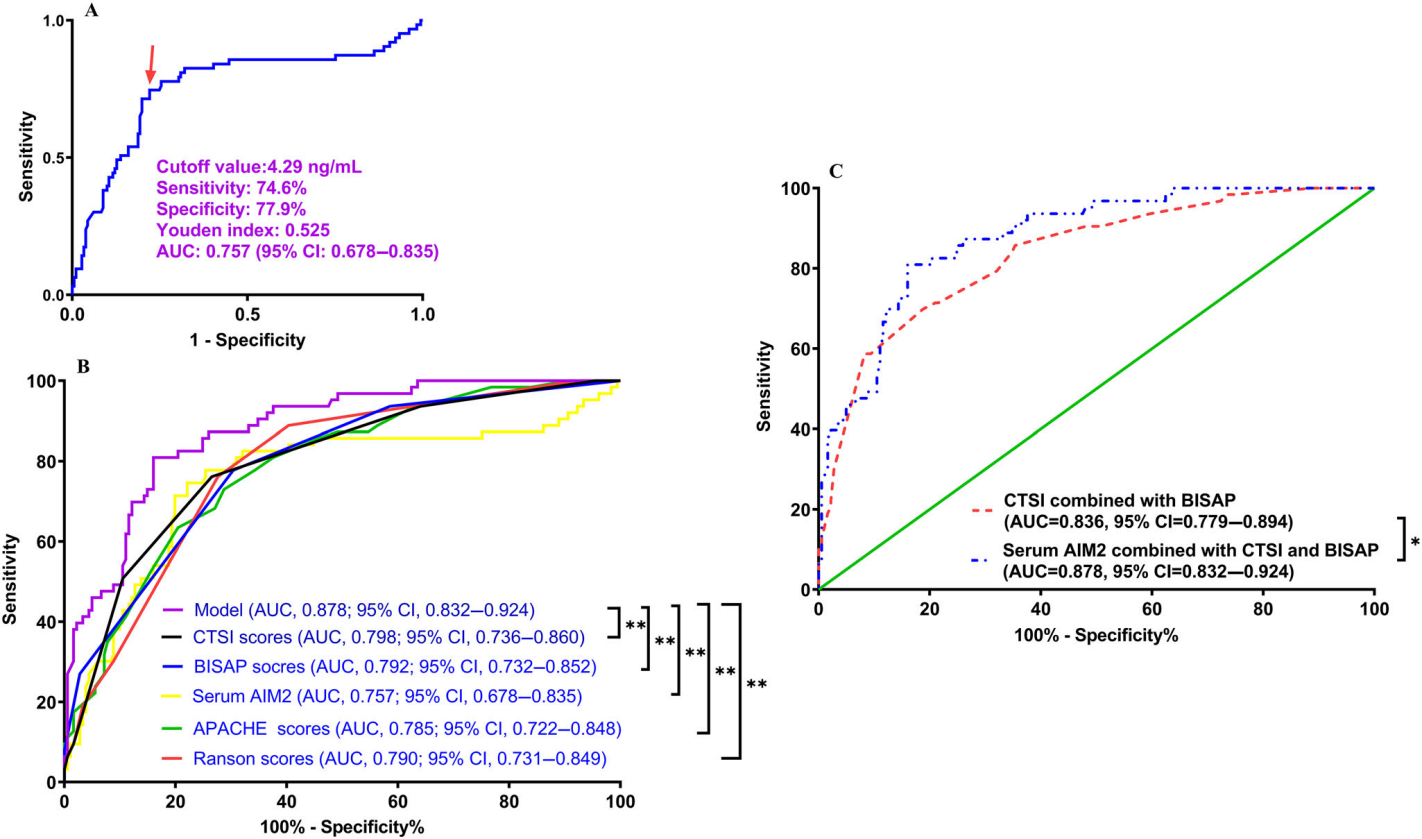
Receiver operating characteristic curve evaluating predictive capability of different indicators for delirium after acute pancreatitis in the study group. Serum absent in melanoma 2 levels effectively predicted delirium after acute pancreatitis (A). Delirium‐prediction model incorporating independent predictors possessed significantly higher predictive ability than the other single variables (all ^**^ *p* < 0.01; B) and the model only encompassing computed tomography severity index scores and bedside index for severity scores (^*^ *p* < 0.05; C). 95% CI, 95% confidence interval; AIM2, absent in melanoma 2; APACHE II, acute physiological and chronic health evaluation II; AUC, area under curve; BISAP, bedside index for severity in acute pancreatitis; CTSI, computed tomography severity index.

In total, 63 patients in the study group and 26 patients in the validation group experienced in‐hospital delirium. The incidence of delirium was not significantly different between the two groups (*p* > 0.05). In the study group, serum AIM2 levels were notably higher in patients with delirium than in those without (*p < *0.001; Figure S1), and were linearly correlated with delirium risk (*p* for nonlinear > 0.05; Figure S2). Serum AIM2 levels predicted delirium with Youden index at 0.525 (Figure 4A). In Table S2, patients presenting with delirium, as opposed to the other remainder, held significantly elevated percentages of high revised Atlanta classification, multiple organ dysfunction syndrome, peripancreatic effusion, pancreatic necrosis, sepsis, acute respiratory failure and acute renal injury, as well as had elevated APACHE II scores, Ranson scores, BISAP scores, CTSI scores, blood C‐reactive protein levels, blood leucocyte count, blood procalcitonin levels, blood glucose levels, and serum AIM2 levels (all *p < *0.05). All these significantly different variables and several clinically relevant indicators were entered into the binary logistic regression model, showing that BISAP scores, CTSI scores, and serum AIM2 levels were independently associated with in‐hospital delirium (all *P < *0.05; Table 4). Using the Hosmer–Lemeshow test, the regression model had satisfactory goodness of fit (*p* = 0.252). Based on subgroup analysis, no significant interactions were demonstrated between serum AIM2 levels and other conventional variables, such as age, sex, and BMI (all *p* interaction > 0.05; Figure S3). Figure S4 shows that the E‐value was 3.15 (95% confidence interval: 2.36‐4.13) for sensitivity analysis.

**TABLE 4:** Multivariate logistic regression analysis of delirium after acute pancreatitis in the study group.

Factors	Odds ratio (95% CI)	*p* value	VIF
Age (years)	1.004 (0.972–1.037)	0.819	1.110
Gender (male/female)	1.144 (0.370–3.535)	0.815	2.010
Body mass index (kg/m^2^)	0.952 (0.846–1.071)	0.414	1.173
Alcohol drinking	1.492 (0.412–5.411)	0.542	2.204
Tobacco smoking	0.392 (0.107–1.436)	0.158	2.330
Hypertension	1.290 (0.498–3.343)	0.600	1.181
Diabetes mellitus	0.908 (0.216–3.816)	0.895	1.284
Admission time after pain (h)	1.536 (0.712–3.312)	0.274	121.222
Sampling time after pain (h)	0.638 (0.304–1.340)	0.235	120.645
Revised Atlanta classification (MAP/ MSAP/ SAP)	1.607 (0.740–3.491)	0.231	2.162
Multiple organ dysfunction syndrome	1.312 (0.467–3.687)	0.606	1.956
Peripancreatic effusion	2.626 (0.952–7.249)	0.062	1.310
Pancreatic necrosis	0.361 (0.105–1.239)	0.105	2.455
Sepsis	1.593 (0.522–4.597)	0.389	1.989
Acute respiratory failure	1.781 (0.622–5.096)	0.282	1.680
Acute renal injury	0.839 (0.224–3.133)	0.794	1.460
Acute physiology and chronic health evaluation II scores	1.034 (0.948–1.129)	0.451	4.416
Ranson scores	1.233 (0.818–1.857)	0.317	5.708
Bedside index for severity in acute pancreatitis	2.389 (1.204–4.741)	0.013	5.738
Computed tomography severity index	1.953 (1.201–3.177)	0.007	3.193
Etiologies (biliary/ alcoholic/hypertriglyceremic/ others)	0.855 (0.535–1.369)	0.515	1.170
Blood C‐reactive protein levels (mg/L)	0.998 (0.987–1.009)	0.718	1.341
Blood leucocyte count (×10^9^/L)	1.018 (0.926–1.118)	0.719	1.426
Blood procalcitonin levels (ng/mL)	1.240 (1.000–1.538)	0.050	1.169
Blood glucose levels (mmol/L)	0.992 (0.912–1.079)	0.850	1.264
Serum absent in melanoma 2 levels (ng/mL)	1.384 (1.071–1.789)	0.013	1.230

Abbreviations: 95% CI, 95% confidence interval; MP, mild acute pancreatitis; MSP, moderately‐severe acute pancreatitis; SP, severe acute pancreatitis; VIF, variance inflation factor.

In Table S3, predictive ability of serum AIM2 levels was substantially superior to those of blood C‐reactive protein levels, blood leucocyte count, blood procalcitonin levels, blood calcium levels, blood creatinine levels, blood urea nitrogen levels, and blood glucose levels (all *p < *0.05). The three independent predictors of delirium in the study group (that is, BISAP scores, CTSI scores, and serum AIM2 levels) were merged to construct nomogram model (Figure S5). The nomogram model was stable (Figure S6), had satisfactory clinical efficacy (Figure S7), and performed the best among numerous variables in terms of discrimination effectiveness (all *p < *0.01; Figure 4B). Combination of BISAP scores and CTSI scores had significantly lower discrimination ability than nomogram model (*p < *0.05; Figure 4C). In Figure S8, when nomogram model was compared to that integrating BISAP scores and CTSI scores, NRI was 0.452 (95% confidence interval, 0.175–0.730; *p = *0.001) and IDI was 0.129 (95% confidence interval, 0.070–0.189; *p < *0.001).

**FIGURE 5:**
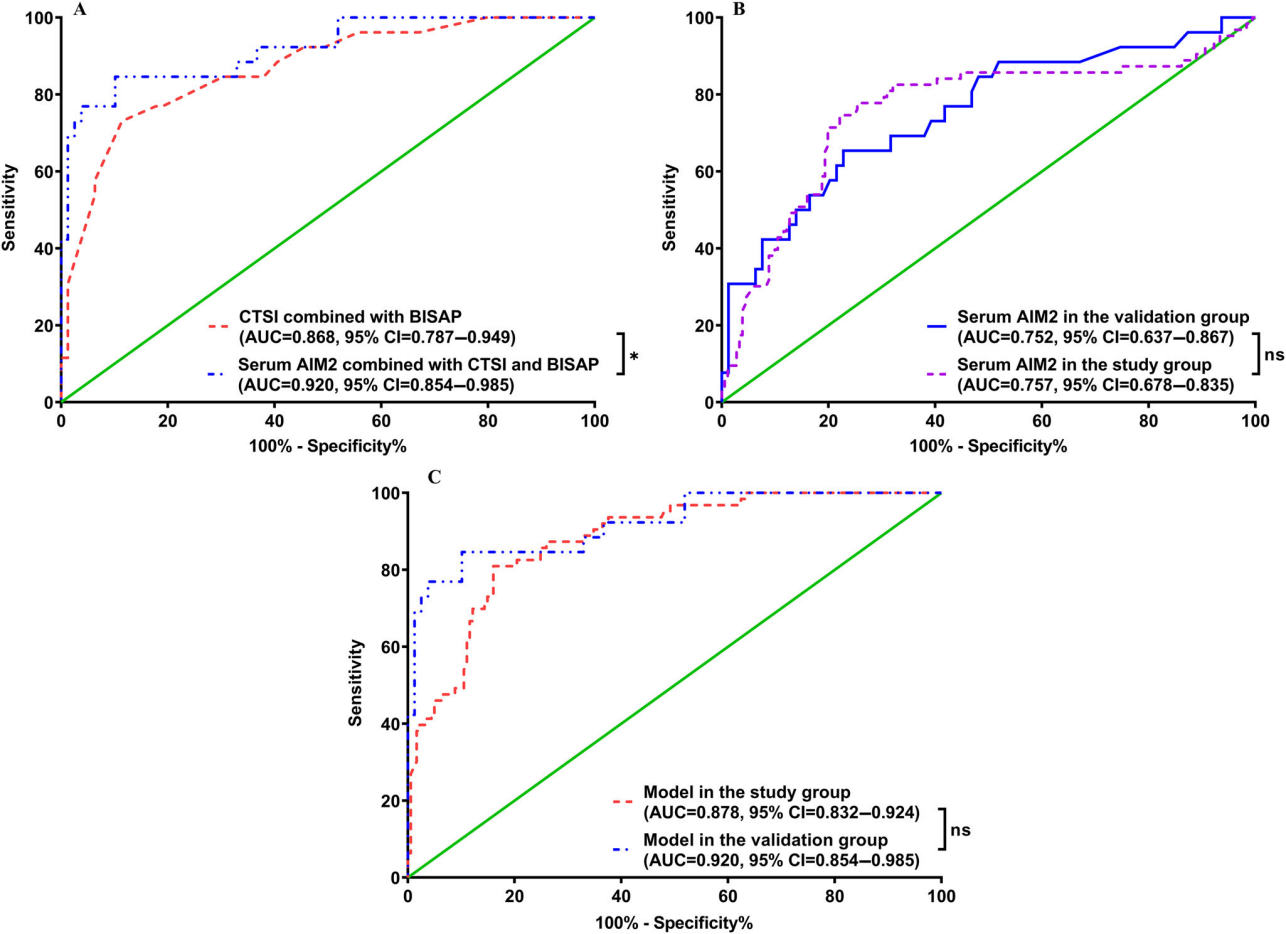
Receiver operating characteristic curve evaluating predictive ability of different metrics for delirium after acute pancreatitis between the study group and validation group. Model integrating serum absent in melanoma 2 levels, computed tomography severity index scores and bedside index for severity in acute pancreatitis scores, in contrast to that only incorporating the latter two metrics, displayed significantly enhanced delirium‐prediction capability in the validation group (^*^ *p* < 0.05; A). Serum absent in melanoma 2 levels showed analogous predictive ability for delirium between the study group and validation group (P>0.05; B). Model integrating serum absent in melanoma 2 levels, computed tomography severity index scores and bedside index for severity in acute pancreatitis scores owned similar predictive capability for delirium between the study group and validation group (*p* > 0.05; C). 95% CI, 95% confidence interval; AIM2, absent in melanoma 2; AUC, area under the curve; BISAP, bedside index for severity in acute pancreatitis; CTSI, computed tomography severity index; ns, non‐significant.

Using data from the validation group, the BISAP scores, CTSI scores, and serum AIM2 levels were consolidated to create nomogram model (Figure S9). The nomogram model was stable (Figure S10) and was clinically valid (Figure S11). Combination of BISAP scores and CTSI scores had substantially declined prediction ability, when compared with nomogram model in the validation group (*p < *0.05; Figure 5A). As delineated in Figure S12, NRI equaled to 0.423 (95% confidence interval, 0.201–0.645; *p < *0.001) and IDI equaled to 0.203 (95% confidence interval, 0.099–0.308; *p < *0.001) when nomogram model was compared to that incorporating BISAP scores and CTSI scores. Moreover, both serum AIM2 levels (*p* > 0.05; Figure 5B) and nomogram model (*p* > 0.05; Figure 5C) possessed similar predictive abilities between study group and validation group.

## 4 Discussion

To the best of our knowledge, there is a paucity of literature available regarding circulating AIM2 levels in AP patients. Some chief findings are that (1) serum AIM2 levels were significantly heightened in patients following AP, as opposed to controls; (2) serum AIM2 levels were independently correlated with APACHE II scores, Ranson scores, BISAP scores, and CTSI scores of patients with AP; (3) serum AIM2 levels, BISAP scores and CTSI scores were the three independent predictors of delirium after AP; and (4) serum AIM2 levels and its combined model owned satisfactory predictive ability, as demonstrated in the study group and validation group. Collectively, serum AIM2 levels and its combined model may be of clinical value for delirium prediction following AP.

Undoubtedly, AP features inflammatory traits in local pancreatic tissues and the whole body, leading to systemic inflammatory response syndrome and resultant multiple organ damage (Mederos et al. 2021; Lankisch et al. 2015). Thus, severity evaluations are not limited to local phenomena, and have been extended to multiple organs and systems throughout the body (Mederos et al. 2021; Lankisch et al. 2015). Inflammasomes are important components of inflammatory modules in the body (Rathinam and Fitzgerald 2016). AIM2, a cytosolic danger‐sensing molecule, functions as an intracellular double‐stranded DNA sensor that recognizes cytosolic DNA derived from damaged cells and subsequently recruits the adaptor protein apoptosis‐associated speck‐like protein containing a CARD, leading to inflammasome assembly and caspase‐1 activation; and activated caspase‐1 promotes the maturation and release of pro‐inflammatory cytokines, including interleukin‐1beta and interleukin‐18, and induces gasdermin D‐mediated pyroptotic cell death (Wang and Yin 2017). Previous experimental studies have demonstrated that AIM2 activation aggravates pancreatic injury, whereas AIM2 deficiency attenuates acinar cell damage, inflammatory cytokine production, and inflammatory cell infiltration in experimental AP models (Kang et al. 2016). Therefore, elevated circulating AIM2 levels observed in our cohort may reflect enhanced inflammasome activation and systemic inflammatory burden during AP progression.

AIM2 has emerged as a promising injury biomarker, as evidenced by a gradually increasing body of data showing that blood AIM2 levels are highly related to severity and poor clinical outcomes in various inflammation‐related disorders such as septicemia (Nasiri and Kariminik 2021), coronary artery disease (Zhang et al. 2022), and stroke‐associated pneumonia (Zhang et al. 2024). Recently, AIM2 was implicated in the pathophysiological processes of AP (Kang et al. 2016). Among the numerous developed severity evaluation tools, APACHE II, Ranson, BISAP, and CTSI are the three strongest determinants of poor prognosis following AP (Zhou et al. 2019; Chauhan et al. 2022; Xu et al. 2023). Currently, serum AIM2 levels are independently correlated with APACHE II, Ranson, BISAP, and CTSI scores, thus supporting the assumption that serum AIM2 may be useful for severity assessment during AP management.

Delirium is increasingly recognized as a manifestation of acute brain dysfunction induced by systemic inflammatory disturbances (Wilson et al. 2020; Keenan and Jain 2022). AP can trigger excessive release of inflammatory mediators, including interleukin‐1beta, interleukin‐6, and tumor necrosis factor‐alpha, maybe resulting in endothelial activation, increased blood–brain barrier permeability, and subsequent neuroinflammatory responses (Beij et al. 2025). In addition, circulating inflammatory signals may activate resident microglia and disrupt neuronal homeostasis, leading to impaired neurotransmission and cognitive dysfunction in the milieu of sepsis (Denver and Cunningham 2025). AIM2 has been implicated in neurological injury through inflammasome‐dependent inflammatory activation and has been shown to contribute to blood–brain barrier disruption, neuronal injury, and neurological deterioration in experimental models (Yuan et al. 2020; Denes et al. 2015; Xu et al. 2021). Therefore, increased serum AIM2 levels may represent not only systemic inflammatory activation but also a biological indicator of susceptibility to inflammation‐associated cerebral dysfunction and delirium (Matsuyama et al. 2020; Ma et al. 2025). Compared with conventional inflammatory biomarkers, such as C‐reactive protein, procalcitonin and blood white cell, AIM2 represents activation of an intracellular danger‐sensing pathway associated with sterile inflammation, tissue injury, and inflammasome‐mediated immune responses (Matsuyama et al. 2020; Ma et al. 2025). This distinction may partially explain why AIM2 demonstrated an independent association with delirium after adjustment for conventional clinical indicators in this study. Moreover, serum AIM2 levels had the high discrimination efficiency for delirium risk after AP; and the predictive ability of serum AIM2 levels were significantly higher than those of blood C‐reactive protein levels, blood leucocyte count, blood procalcitonin levels, blood calcium levels, blood creatinine levels, blood urea nitrogen levels and blood glucose levels in this cohort of AP patients. Also, a nomogram model was built based on the three independent predictors of delirium after AP. Discrimination efficacy of serum AIM2 levels and nomogram model was validated in the validation group. Therefore, serum AIM2 may be a potential predictor of in‐hospital delirium following AP and a combined model containing serum AIM2 levels may be able to discriminate delirium risk following AP.

There are two limitations in this study. First, infection status may be an important potential confounder of delirium occurrence in the scenario of AP. In our study, sepsis was included among the clinical variables associated with serum AIM2 levels and delirium subsequent to AP in the univariate analysis, but not existent during multivariable modeling, therefore supporting the conception that serum AIM2 levels and delirium may not be relevant to infection per se, but its secondary reactions. However, other infection‐related clinical manifestations were not collected and analyzed in the current study, the assumption should be further verified. Second, from a clinical perspective, our findings suggest that serum AIM2 may serve as an early risk stratification biomarker rather than an independent diagnostic criterion for delirium. Patients presenting with elevated AIM2 levels after admission may represent a subgroup with enhanced systemic inflammatory activation and increased susceptibility to cerebral dysfunction. In clinical practice, these patients may benefit from intensified surveillance, including repeated delirium screening using validated tools such as CAM, optimization of modifiable risk factors, and early implementation of non‐pharmacological delirium prevention strategies. Nevertheless, the proposed AIM2 cutoff value should be interpreted cautiously because it was derived from a single cohort. Before routine clinical implementation, external validation studies and prospective intervention trials are required to determine whether AIM2‐guided monitoring strategies can effectively reduce delirium incidence and improve patient outcomes.

## 5 Conclusions

Significantly elevated serum AIM2 levels after AP are independently correlated with APACHE II, Ranson, CTSI, and BISAP scores, and are independently predictive of in‐hospital delirium. The delirium‐prediction model integrating serum AIM2 levels performed well. Therefore, serum AIM2 may be a useful biomarker for effectively assessing AP severity and predicting in‐hospital delirium following AP.

## Author Contributions

**Shiyang Dong**: conceptualization, investigation, validation, writing – original draft, writing – review and editing. **Bo Zhang**: conceptualization, methodology, data curation, investigation, validation, formal analysis, supervision, writing – original draft, writing – review and editing. **Yidong Jing**: conceptualization, software, validation, project administration, writing – original draft, writing – review and editing. **Shaojun Zhang**: conceptualization, formal analysis, visualization, project administration, writing – original draft, writing – review and editing. **Zihao Tang**: software, investigation, validation, formal analysis, visualization, project administration, writing – original draft. **Jianye Wu**: conceptualization, validation, formal analysis, visualization, project administration, writing – original draft. **Yujun Rao**: conceptualization, methodology, investigation, validation, formal analysis, writing – review and editing.

## Funding

The authors have nothing to report.

## Conflicts of Interest

The authors declare no conflicts of interest.

## Supporting information




**Supporting File 1**: brb371712‐sup‐0001‐SuppMat.docx

## Data Availability

Data supporting the findings of this study are available upon request from the corresponding author. The data were not publicly available due to privacy or ethical restrictions.
