# Supplementary material for: Serum AIM2 as a Prognostic Biomarker for Disease Severity and Delirium in Acute Pancreatitis: A Multicenter Prospective Cohort Study
Source: Brain Behav. 2026 Aug 20;16(8):e71712. doi: 10.1002/brb3.71712 (PMC13493696; doi:10.1002/brb3.71712)
Supplement: Supplementary file 1 [file BRB3-16-e71712-s001.docx]

Supplementary Table 1 Factors correlated with serum absent in melanoma 2 levels after acute pancreatitis in the study group

| Factors | ρ | P value |
| --- | --- | --- |
| Age (years) | 0.120 | 0.061 |
| Gender (male/female) | -0.110 | 0.086 |
| Body mass index (kg/m^2^) | 0.097 | 0.130 |
| Alcohol drinking | -0.048 | 0.454 |
| Tobacco smoking | -0.035 | 0.588 |
| Hypertension | -0.031 | 0.625 |
| Diabetes mellitus | -0.031 | 0.635 |
| Admission time after pain (h) | 0.103 | 0.108 |
| Sampling time after pain (h) | 0.103 | 0.107 |
| Revised Atlanta classification (MAP/ MSAP/ SAP) | 0.453 | <0.001 |
| Multiple organ dysfunction syndrome | 0.239 | <0.001 |
| Peripancreatic effusion | 0.180 | 0.005 |
| Pancreatic necrosis | 0.237 | <0.001 |
| Sepsis | 0.151 | 0.018 |
| Acute respiratory failure | 0.127 | 0.047 |
| Acute renal injury | 0.235 | <0.001 |
| Acute physiology and chronic health evaluation II scores | 0.607 | <0.001 |
| Ranson scores | 0.609 | <0.001 |
| Bedside index for severity in acute pancreatitis | 0.605 | <0.001 |
| Computed tomography severity index | 0.628 | <0.001 |
| Etiologies (biliary/ alcoholic/hypertriglyceremic/ others) | -0.050 | 0.433 |
| Blood C-reactive protein levels (mg/L) | 0.337 | <0.001 |
| Blood leucocyte count (×10^9^/L) | 0.193 | 0.002 |
| Blood procalcitonin levels (ng/mL) | 0.164 | 0.010 |
| Blood calcium levels (mmol/L) | -0.095 | 0.141 |
| Blood glucose levels (mmol/L) | 0.229 | <0.001 |
| Blood creatinine levels (μmol/L) | 0.154 | 0.016 |
| Blood urea nitrogen levels (mg/dL) | 0.110 | 0.086 |

The Spearman test was used. MP denotes mild acute pancreatitis; MSP, moderately-severe acute pancreatitis; SP, severe acute pancreatitis.

Supplementary Table 2 Factors associated with delirium after acute pancreatitis in the study group

| Factors | Presence of delirium (n=63) | Absence of delirium (n=181) | P value |
| --- | --- | --- | --- |
| Age (years) | 50 (44-59) | 48 (39-59) | 0.422 |
| Gender (male/female) | 34/29 | 109/72 | 0.385 |
| Body mass index (kg/m^2^) | 25.2 (21.3-27.2) | 25.0 (22.3-27.4) | 0.570 |
| Alcohol drinking | 19 (30.2%) | 60 (33.1%) | 0.662 |
| Tobacco smoking | 19 (30.2%) | 70 (38.7%) | 0.227 |
| Hypertension | 16 (25.4%) | 46 (25.4%) | 0.998 |
| Diabetes mellitus | 7 (11.1%) | 25 (13.8%) | 0.584 |
| Admission time after pain (h) | 14.3 (9.5-17.8) | 13.5 (8.9-17.8) | 0.781 |
| Sampling time after pain (h) | 15.3 (11.2-19.4) | 14.8 (10.3-19.2) | 0.864 |
| Revised Atlanta classification (MAP/ MSAP/ SAP) | 4/31/28 | 93/58/30 | <0.001 |
| Multiple organ dysfunction syndrome | 27 (42.9%) | 26 (14.4%) | <0.001 |
| Peripancreatic effusion | 52 (82.5%) | 99 (54.7%) | <0.001 |
| Pancreatic necrosis | 23 (36.5%) | 29 (16.0%) | 0.001 |
| Sepsis | 24 (38.1%) | 27 (14.9%) | <0.001 |
| Acute respiratory failure | 23 (36.5%) | 26 (14.4%) | <0.001 |
| Acute renal injury | 12 (19.0%) | 9 (5.0%) | 0.001 |
| Acute physiology and chronic health evaluation II scores | 16 (12-20) | 8 (4-14) | <0.001 |
| Ranson scores | 3 (3-4) | 1 (1-3) | <0.001 |
| Bedside index for severity in acute pancreatitis | 3 (3-4) | 2 (1-3) | <0.001 |
| Computed tomography severity index | 4 (3-4） | 2 (1-3） | <0.001 |
| Etiologies (biliary/ alcoholic/hypertriglyceremic/ others) | 24/23/12/4 | 53/73/39/16 | 0.611 |
| Blood C-reactive protein levels (mg/L) | 42.9 (35.2-83.8) | 33.6 (23.2-60.5) | 0.001 |
| Blood leucocyte count (×10^9^/L) | 15.7 (11.7-18.9) | 13.2 (10.5-16.2) | 0.011 |
| Blood procalcitonin levels (ng/mL) | 2.9 (1.8-5.2) | 2.4 (1.8-3.6) | 0.019 |
| Blood calcium levels (mmol/L) | 2.0 (1.8-2.1) | 2.1 (1.9-2.2) | 0.163 |
| Blood glucose levels (mmol/L) | 11.6 (8.7-18.8) | 10.4 (7.8-14.1) | 0.002 |
| Blood creatinine levels (μmol/L) | 116.2 (105.5-144.2) | 112.6 (91.1-137.9) | 0.129 |
| Blood urea nitrogen levels (mg/dL) | 12.9 (10.9-16.9) | 12.6 (9.4-15.0) | 0.078 |
| Serum absent in melanoma 2 levels (ng/mL) | 5.3 (4.3-6.1) | 3.5 (3.0-4.2) | <0.001 |

Qualitative variables were presented as counts (percentages) and quantitative variables were reported as medians (percentiles 25th-75th). The statistical methods encompassed the chi-square test, Fisher exact test and Mann-Whitney U test. MP denotes mild acute pancreatitis; MSP, moderately-severe acute pancreatitis; SP, severe acute pancreatitis.

Supplementary Table 3 Predictive abilities of multiple metrics for delirium after acute pancreatitis in the study group

| Factors | AUC (95% CI) | P value |
| --- | --- | --- |
| Blood C-reactive protein levels (mg/L) | 0.639 (0.567 to 0.711) | 0.017 |
| Blood leucocyte count (×10^9^/L) | 0.606 (0.523 to 0.689) | 0.010 |
| Blood procalcitonin levels (ng/mL) | 0.599 (0.509 to 0.689) | 0.006 |
| Blood calcium levels (mmol/L) | 0.560 (0.478 to 0.642) | <0.001 |
| Blood glucose levels (mmol/L) | 0.629 (0.546 to 0.711) | 0.016 |
| Blood creatinine levels (μmol/L) | 0.564 (0.485 to 0.643) | 0.001 |
| Blood urea nitrogen levels (mg/dL) | 0.574 (0.492 to 0.657) | <0.001 |
| Serum absent in melanoma 2 levels (ng/mL) | 0.757 (0.678 to 0.835) | Reference |

Z test was adopted for comparison of area under curve. AUC indicates area under receiver operating characteristic curve; 95% CI, 95% confidence interval.

**Figure legends**

**Supplementary Figure 1**

Serum absent in melanoma 2 levels of patients with delirium after acute pancreatitis in the study group.

Serum absent in melanoma 2 levels were substantially higher in patients with delirium than in those without delirium (P<0.001).

AIM2 stands for absent in melanoma 2.

**Supplementary Figure 2**

Linearity relationship between serum absent in melanoma 2 levels and delirium probability following acute pancreatitis in the study group.

Under restricted cubic spline, serum absent in melanoma 2 levels were linearly correlated with delirium likelihood after acute pancreatitis (P for nonlinear > 0.05).

AIM2 indicates absent in melanoma 2.

**Supplementary Figure 3**

Subgroup analysis determining moderating effects in delirium analysis following acute pancreatitis in the study group.

Conventional variables did not show interactional effects with serum absent in melanoma 2 levels for association with delirium after acute pancreatitis (all P interaction > 0.05).

OR signifies odds ratio; 95% CI, 95% confidence interval.

**Supplementary Figure 4**

E-value for sensitivity analysis in the study group.

E-value was visualized.

**Supplementary Figure 5**

Nomogram predicting delirium after acute pancreatitis in the study group.

Nomogram model encompassed serum absent in melanoma 2 levels, computed tomography severity index, and bedside index for severity in acute pancreatitis.

AIM2 indicates absent in melanoma 2; CTSI, computed tomography severity index; BISAP, bedside index for the severity in acute pancreatitis.

**Supplementary Figure 6**

Calibration curve assessing nomogram model in the study group.

Nomogram model possessed a good calibration for predicting delirium after acute pancreatitis.

**Supplementary Figure 7**

Decision curve appraising nomogram model in the study group.

The nomogram model showed clinical validity.

APACHE II means Acute Physiological and Chronic Health Evaluation II; CTSI, computed tomography severity index; BISAP, bedside index for severity in acute pancreatitis; AIM2, absent in melanoma 2.

**Supplementary Figure 8**

Classification plot evaluating nomogram model in the study group.

The standard model encompassed computed tomography severity index and bedside index for severity in acute pancreatitis. Th new model, that is nomogram model, displayed significant net reclassification improvement and integrated discrimination improvement.

**Supplementary Figure 9**

Nomogram predicting delirium following acute pancreatitis in the validation group.

Nomogram model was composed of serum absent in melanoma 2 levels, computed tomography severity index, and bedside index for severity in acute pancreatitis.

AIM2 indicates absent in melanoma 2; CTSI, computed tomography severity index; BISAP, bedside index for the severity in acute pancreatitis.

**Supplementary Figure 10**

Calibration curve appraising nomogram model in the validation group.

Nomogram model had a satisfactory correctness for delirium prediction after acute pancreatitis.

**Supplementary Figure 11**

Decision curve appraising nomogram model in the validation group.

The nomogram model demonstrated clinical effectiveness.

APACHE II means Acute Physiological and Chronic Health Evaluation II; CTSI, computed tomography severity index; BISAP, bedside index for severity in acute pancreatitis; AIM2, absent in melanoma 2.

**Supplementary Figure 12**

Classification plot assessing nomogram model in the validation group.

The standard model was comprised of computed tomography severity index and bedside index for severity in acute pancreatitis. Th new model, that is nomogram model, exhibited significant net reclassification improvement and integrated discrimination improvement.

Supplementary Figure 1

Supplementary Figure 2

Supplementary Figure 3

Supplementary Figure 4

Supplementary Figure 5

Supplementary Figure 6

Supplementary Figure 7

Supplementary Figure 8

Supplementary Figure 9

Supplementary Figure 10

Supplementary Figure 11

Supplementary Figure 12
